# Cloning and Expression of Multiple Cytochrome P450 Genes: Induction by Fipronil in Workers of the Red Imported Fire Ant (*Solenopsis invicta* Buren)

**DOI:** 10.1371/journal.pone.0150915

**Published:** 2016-03-16

**Authors:** Baizhong Zhang, Lei Zhang, Rukun Cui, Xinnian Zeng, Xiwu Gao

**Affiliations:** 1 Key Laboratory of Natural Pesticide and Chemical Biology, Ministry of Education, South China Agricultural University, Guangzhou 510642, P.R. China; 2 Department of Entomology, China Agricultural University, Beijing 100193, P.R. China; 3 College of Natural Resources and Environment, Henan Institute of Science and Technology, Xinxiang 453003, P.R. China; Institute of Zoology, Chinese Academy of Sciences, CHINA

## Abstract

Both exogenous and endogenous compounds can induce the expression of cytochrome P450 genes. The insect cytochrome P450 genes related to insecticide resistance are likely to be expressed as the “first line of defense” when challenged with insecticides. In this study, four cytochrome P450 genes, *SinvCYP6B1*, *SinvCYP6A1*, *SinvCYP4C1*, and *SinvCYP4G15*, were firstly isolated from workers of the red imported fire ant (*Solenopsis invicta*) through rapid amplification of cDNA ends (RACE) and sequenced. The fipronil induction profiles of the four cytochrome P450 genes and the two previously isolated *CYP4AB1* and *CYP4AB2* were characterized in workers. The results revealed that the expression of *SinvCYP6B1*, *SinvCYP6A1*, *CYP4AB2*, and *SinvCYP4G15*, increased 1.4-fold and 1.3-fold more than those of acetone control, respectively, after 24 h exposure to fipronil at concentrations of 0.25 μg mL^−1^ (median lethal dose) and 0.56 μg mL^−1^ (90% lethal dose), while no significant induction of the expression of *CYP4AB1* and *SinvCYP4C1* was detected. Among these genes, *SinvCYP6B1* was the most significantly induced, and its maximum expression was 3.6-fold higher than that in acetone control. These results might suggest that multiple cytochrome P450 genes are co-up-regulated in workers of the fire ant through induction mechanism when challenged with fipronil. These findings indicated that cytochrome P450 genes play an important role in the detoxification of insecticides and provide a theoretical basis for the mechanisms of insecticide metabolism in the fire ant.

## Introduction

The red imported fire ant (RIFA; *Solenopsis invicta*, Hymenoptera, Formicidae, Myrmicinae, Solenopsis) is recognized as the most invasive and destructive alien species because of the complexity of its diet and its aggressive nature, rapid reproduction and strong competitive ability [1]. Many methods have been used to control the fire ant, but none have permanently eradicated this species from an area. To date, chemical control has been the most effective measure, but there is a need for a more effective control agent [2,3] because the long-term use of chemicals for insect control can lead to an unexpected rebound and increased tolerance in colonies of the fire ant [4]. Fipronil, a phenylpyrazole insecticide, exhibits neurotoxic activity by blocking the GABA-regulated chloride channels of neurons [5,6]. Formulation of fipronil into granules or bait has been demonstrated to be effective against RIFA [7]. These insecticides are widely used to control crop pests, and the development of resistance among many pests has been reported [8–12]. However, the tolerance of the fire ant to insecticides has not been reported.

Similar to most other insects, social insects (e.g., ants and honeybees) rely in part on a suite of detoxification enzymes to metabolize naturally occurring xenobiotics and pesticides. Cytochrome P450 monooxygenases (P450s) are a major component of these enzyme suites [13]. P450s are central to the tolerance of pesticides and the evolved resistance to pesticides in many pest insects [14], as P450s play a role in pesticide detoxification [15,16]. Another characteristic of certain insect P450 genes is that both exogenous and endogenous compounds induce their expression [17]. The induction and activity of P450 in insects are proposed to be involved in the adaptation of insects to the environment and in the development of insecticide resistance [18]. The induction of P450 is dose- and time-dependent, and P450 can be induced only when the inducing agent reaches a specific concentration after a specific period of time [19]. P450 genes have been induced in many insects [17]. Recent studies examining the induction of P450 by exogenous compounds have focused on plant secondary metabolites, herbicides and pyrethroid insecticides [20–22]. Nevertheless, the induction of P450s by insecticides in insects is less well understood, particularly in the fire ant.

The present study focused on isolating full-length cDNA sequences corresponding to the available expressed sequence tags (ESTs), characterizing the expression profiles of these P450 genes in workers of the fire ant, and determining the response of the P450 genes to fipronil treatment in workers; the possible roles of these genes are discussed. These findings will provide a direct and powerful basis for assessing the tolerance to insecticides and determining the rational usage of insecticides to control this social pest.

## Materials and Methods

### Insects

Colonies of the red imported fire ant used in the study were collected from the lawn of the teaching and experimental campus of South China Agricultural University, Guangzhou, China, which issued permission for the performance of the study at this site. Collection was performed according to the method of Kuriachan et al. [23]. The colonies were transferred into a 30-L plastic box (50 x 30 x 20 cm) coated with talcum powder to prevent escape. The fire ant were fed with a 10% sugar solution and were maintained at 27±1°C, 70–90% relative humidity, with a 16:8 h light:dark photoperiod. Healthy workers at an average body weight of 1.6 mg were selected for test.

### Insecticides and chemicals

Fipronil (95%, technical-grade powder) was obtained from the Wuxi Ruize Pesticide Company, Ltd. TRIzol reagent was purchased from Invitrogen (Shanghai, China); Taq DNA polymerase and the DNA Marker DL 2000 were purchased from the Sangon Company (Shanghai, China); agarose, DNase I, and SYBR Green I were purchased from the TaKaRa Company (Dalian, China); and the MEGAscript® RNAi kit was purchased from Ambion (Austin, TX). All chemicals were reagent grade.

### Insecticide treatment

Contact toxicity to workers was evaluated in the laboratory. The test insecticide was dissolved in an appropriate amount of acetone and then serially diluted [24]. First, 1.00 mL of the liquid solution was pipetted into disposable plastic cups (the top caliber, bottom diameter, and height were 6.6, 4.4, and 6.8 cm, respectively) with an application area of approximately 72.85 cm^2^. Second, the cups were shaken to ensure even coating until the acetone had completely evaporated; subsequently, the cups were coated with an appropriate amount of talcum powder in the caliber (on the inside surface) to prevent workers from climbing out. Finally, approximately 200 workers for each treatment were exposed to fipronil at median lethal dose and 90% lethal dose for 12, 24, 36, 48, 60, and 72 h to determine the effects of fipronil on the expression of P450 genes. The workers survived were collected for the measurement of the expression of P450 genes. Acetone exposures were considered as control treatments. Three replicates were employed per concentration. The workers were fed with honey water (5%) on a small cotton ball. Five workers for each treatment were sampled. The samples were immediately frozen in liquid nitrogen and stored at -80°C for total RNA isolation.

### Primer design

The PCR primers and the 3’ and 5’ RACE primers were designed using Primer 5.0 (Premier Biosoft International, Palo Alto, CA). These primers were used for the cloning of the four target genes fromthe fire ant. The primers used for quantitative real-time PCR (qRT-PCR) were designed using primer3 (http://primer3.ut.ee/) and were based on the sequences published in NCBI for the clones *12H3* (Accession No: EH413135), *24E8* (Accession No: EH413135), *C423* (Accession No: EH413754.1), *9E4* (Accession No: EH413020.1), *CYP4AB1* (Accession No: AY345970), *CYP4AB2* (Accession No: AY345971), and *18S rRNA* (Accession No: AY334566).

### RNA extraction and cDNA preparation

Total RNA was extracted from 150 mg flash-frozen individuals of the fire ant (whole body) using the TRIzol kit (Invitrogen, Carlsbad, CA) according to the manufacturer’s instructions. The first-strand cDNA was synthesized using a PrimeScript kit (Takara Biotechnology, Dalian, China). PCR thermal cycling was performed as follows: an initial denaturation at 94°C for 3 min followed by 35 cycles at 94°C for 30 s, 60–45°C (depending on the primer pair) for 30 s, and 72°C for 1–3 min (determined by the length of the amplified fragment) with an additional polymerization step at 72°C for 10 min. The PCR products were cloned and sequenced using the pGEM-T Easy Vector System II (Promega, Madison, WI).

### Rapid amplification of cDNA ends (RACE)

The 3’ and 5’ RACE first-strand cDNAs were synthesized, and the PCR system was designed according to the instructions provided with the Smart^TM^ Race cDNA Amplification Kit (Clontech). Based on the sequences published in NCBI for the clones 12H3 (Accession No: EH413135), 24E8 (Accession No: EH413135), C423 (Accession No: EH413754.1), and 9E4 (Accession No: EH413020.1), 16 gene-specific primers (GSPs) were designed to amplify the full-length cDNAs. The designed primers are presented in Table 1. Thermal cycling was performed using touchdown PCR as follows: 5 cycles at 94°C for 30 s and 72°C for 3 min; 5 cycles at 94°C for 30 s, 70°C for 30 s, and 72°C for 3 min; and 27 cycles at 94°C for 30 s, 68°C for 30 s, and 72°C for 3 min. The products of 3' and 5' RACE were cloned into the pGEM-T Easy Vector and sequenced.

**Table 1:** Sequences of the primers used for cloning and measuring the relative expression of the target genes.

Purpose/primer name	Sequence (5´–3´)
cDNA isolation (real-time PCR (RT-PCR)	
12H3 (*SinvCYP6B1*)	Sense: GTTATCTCACCGAGCGAGCT
	Antisense: CACCTTGGTCCGCTACTTTC
*SinvCYP6B1*	Sense: ACGACCCAAACACAGCAGCA
	Antisense: TTTTTATGGAGGCTTAGAGAG
9E4 (*SinvCYP4C1*)	Sense: TCTGTCCTCCGATCTCAT
	Antisense: TAATCCCAGTCCCTAACC
*SinvCYP4C1*	Sense: CAGTGGTATCAACGCAGAG
	Antisense: GATTGGGACAAATCGTGT
24E8 (*SinvCYP6A1*)	Sense:AACAGTTATACTTAAAAACACGCTAAA
	Antisense: ACCTTGCCCGATACAATTTC
*SinvCYP6A1*	Sense: ATCACGGGTATCACAATTGC
	Antisense: ATCCTCTTAGGTGATTTTGC
C423 (*SinvCYP4G15*)	Sense: AGCGGTTCGAGTTTGATG
	Antisense: ACGAGGAGATTTGGGAGG
*SinvCYP4G15*	Sense: TAGAACCAGTAACAACACCC
	Antisense: ATCGTCCCTTTATCTCAGAA
5′ and 3′ cDNA end isolation	
(rapid amplification of cDNA ends)	
#12H3 (*SinvCYP6B1*)	5′GPS1: TCGGTCGTGTATTTAGCAGA
	5′GPS2: TCGGTTTGAGGCAGGAAGTA
	3′GPS3: AAGACACCCCTTCTCATT
#9E4 (*SinvCYP4C1*)	5′GPS1: TGTCAGCGTTCACCATCC
	5′GPS2: CTTCCTCCCAAATCTCCT
	3′GPS3: TGTCAGCGTTCACCATCC
	3′GPS4: GGTTAGGGACTGGGATTA
	3′GPS5: AAGCAACACAAAGGAACATC
	3′GPS6: AATGGCACAAAAGGCGAAAGA
#24E8 (*SinvCYP6A1*)	5′GPS1: GCTTCGTGTAATGTCCGTCA
	5′GPS2: CGAGATACATTACTGGTGGA
	3′GPS3: CCCGTGGTTCTACAAGAACTTCGGACAT
#C423 (*SinvCYP4G15*)	5′GPS1: CCAGCACTGAAAGGAATG
	5′GPS2: CTTCCTCCCAAATCTCCT
	3′GPS3: CCAGCACTGAAAGGAATG
	3′GPS4: CTTCCTCCCAAATCTCCT
Expression analysis	
(Quantitative real-time PCR (qRT-PCR)	
*18S rRNA*	Sense: GAATTCCCAGTAAGCGCGAG
	Antisense: GTCATCTTCCCGGCAACATC
*SinvCYP6B1*	Sense: TGACGGACATTACACGAAGC
	Antisense: CCAATTCGTACATTGCATGG
*SinvCYP6A1*	Sense: CCATGTTCGCTGATAGAGGA
	Antisense: CACCTTGGTCCGCTACTTTC
*CYP4AB1*	Sense: AGAACGTGGGCTTTCTTTGA
	Antisense: GCCATTTGGAACCTCCACTA
*CYP4AB2*	Sense: GGCATTTACGGCGAAAGATA
	Antisense: AGTTCCCATGGCAGTTTCAC
*SinvCYP4C1*	Sense: TTTTGTCAGCGTTCACCATC
	Antisense: AGCATCTTTCGCCTTTTGTG
*SinvCYP4G15*	Sense: GTCTGTCGCTGTCACCAAAA
	Antisense: ACTACGGCAGCTGGTTCAAG

### Sequence analysis

The sequenced cDNA fragments were assembled into a consensus sequence that contained the complete open reading frame. The molecular weights and the pIs were predicted using the ExPASy website (http://web.expasy.org/compute-pi/). Amino acid alignments with other sequences and protein lengths were determined with the DNAMAN v. 6.03 program. The SignalP 4.0 server (http://www.cbs.dtu.dk/services/SignalP/) and Geneious (v. 4.8.4) were used for the predictions of the signal peptides and the possible transmembrane regions, respectively [25]. In total, the complete cDNAs of the insect CYP4 and CYP6 families, which were obtained from GenBank (http://www.ncbi.nlm.nih.gov/), were used to construct the phylogenetic tree, based on amino acid sequences with the software programs CLUSTALX 2.0 and MEGA 5.0 [26]. The phylogenetic analysis was performed using maximum parsimony. The sample variance of the distance values was estimated from 10,000 bootstrap replicates of the alignment columns.

### Quantitative real-time PCR (qRT-PCR)

Total RNA extraction from workers was performed as described above, and the resulting total RNA was re-suspended in nuclease-free water and was quantified on a NanoDrop 2000 spectrophotometer (Thermo Scientific, Wilmington, DE). First-strand cDNAs were synthesized with 1.0 μg/μl total RNA using the PrimeScript® RT reagent kit (Takara Biotech, Dalian, China) according to the manufacturer’s protocol.

Quantitative real-time PCR (qRT-PCR) reactions were performed in a 20-μL mixture that contained 1.0 μL of cDNA, 10 μL of SYBR Green qRT-PCR SuperMix-UDG, 0.15 μL of each primer and 8.7 μL of H_2_O according to the instructions provided with the Invitrogen Platinum SYBR Green qRT-PCR SuperMix-UDG kit. The primers designed for this study are presented in Table 1. The amplification efficiency of the target genes and the housekeeping gene (*18S rRNA*) was estimated using E = 10^−1^/slope – 1, where the slope is derived from the plot of the cycle threshold (C_t_) values versus the log of the serially diluted template concentrations. The optimized qRT-PCR program consisted of an initial step at 50°C for 2 min and 94°C for 2 min, followed by 50 cycles at 94°C for 15 s and 60°C for 30 s. After the cycling protocol, melting curves were obtained by increasing the temperature from 60°C to 95°C (0.2°C s^−1^) to denature the double-stranded DNA. The qRT-PCR amplifications were conducted in 96-well plates. The assays were run on an ABI 7500 system using SDS v.1.4 software (Applied Biosystems). Quantification of the transcript levels of SinvCYP6B1, SinvCYP6A1, SinvCYP4C1, SinvCYP4G15, CYP4AB1, and CYP4AB2 mRNAs was performed using the comparative 2^–ΔΔCT^ method [27].

### Statistical analyses

The analyses were performed in EXCEL (2010) and Polo (Probit and Logit Analysis) (LeOra Software Company). To compare the mRNA expression levels of the six P450 genes (*SinvCYP6B1*, *SinvCYP6A1*, *SinvCYP4C1*, *SinvCYP4G15*, *CYP4AB1*, and *CYP4AB2*) from workers of the fire ant, the data were analyzed using Student’s t test (p<0.05) with SPSS (15.0) or using one-way ANOVA and Tukey’s test (p<0.05) with InStat v. 3.0 (GraphPad Software, San Diego, CA).

## Results

### Contact toxicity of fipronil against workers

The contact toxicity of fipronil against workers is relatively high, with LC_50_ and LC_90_ values of 0.25 μg mL^−1^ and 0.56 μg mL^−1^, respectively.

Time-dependent mortality in workers treated with fipronil at the calculated concentrations of LC_50_ and LC_90_ (0.25 μg mL^−1^ and 0.56 μg mL^−1^) was shown in Table 2. The mortalities for LC_50_ ranged from 12.7% to 83.8%, indicating dosage suitability for induction study of gene expression in the duration of 12–72 h exposure, while the mortalities for LC_90_ reached 100% after 48 h exposures.

**Table 2:** Time-dependent mortality of workers exposed to fipronil at 0.25 μg mL^−1^ and 0.56 μg mL^−1^ after 12, 24, 36, 48, 60, and 72 h. Data are presented as the mean ± standard error (S.E.) of three independent replicates. Different letters after the standard errors indicate significant differences among treatments based on ANOVA followed by Tukey’s multiple comparison test (p<0.05) for the same time point.

Time after exposure		Mortality %	
to fipronil	Control	Treatment	Treatment
	(0 μg mL^−1^)	(0.25 μg mL^−1^)	(0.56 μg mL^−1^)
12 h	1.7±0.6 c	12.7±2.5 b	41.7±5.7 a
24 h	3.3±2.1 c	30.7±4.1 b	86.3±6.0 a
36 h	4.3±1.2 c	50.7±1.5 b	98.3±10.1 a
48 h	6.3±0.6 c	64.7±5.7 b	100±0.0 a
60 h	8.0±2.5 c	72.6±9.5 b	100±0.0 a
72 h	9.3±2.5 c	83.8±9.5 b	100±0.0 a

### Identification and characterization of four cytochrome P450 genes from workers

The sequences of the cDNA fragments amplified using the SMART RACE technique perfectly overlapped with the sequences of the clones #12H3 (SinvCYP6B1), #24E8 (SinvCYP6A1), #9E4 (SinvCYP4C1), and #C423 (SinvCYP4G15), which indicated that the fragments were the 5' and 3' ends of the putative P450 genes. The cDNA sequences of the full-length clones #12H3 (SinvCYP6B1), #24E8 (SinvCYP6A1), #9E4 (SinvCYP4C1), and #C423 (SinvCYP4G15) had open reading frames (ORFs) of 1497, 1500, 1560, and 1590 bp, respectively, which encoded putative amino acid sequences of 499, 500, 520, and 530 amino acids, respectively. The putative amino acid sequences shared 77%, 58%, 58%, and 75% identity with *Acromyrmex echinatior CYP6A1* (Accession No: EGI67003.1), *Harpegnathos saltator CYP6A14* (Accession No: EGI66978.1), *Camponotus floridanus CYP4C1* (Accession No: EFN68667.1), and *Acromyrmex echinatior CYP4G15* (Accession No: EGI64338.1), respectively; therefore, the sequences were named *SinvCYP6B1* (Accession No: KF547037), *SinvCYP6A1* (Accession No: KF547038), *SinvCYP4C1* (Accession No: KM006494), and *SinvCYP4G15* (Accession No: KM006495), respectively, according to the P450 nomenclature committee (D. Nelson, personal communication). These sequences have molecular masses of 57.65, 58.08, 60.00, and 61.39 kDa and predicted pIs of 8.59, 8.20, 7.06, and 8.10, respectively.

**Fig 1:**
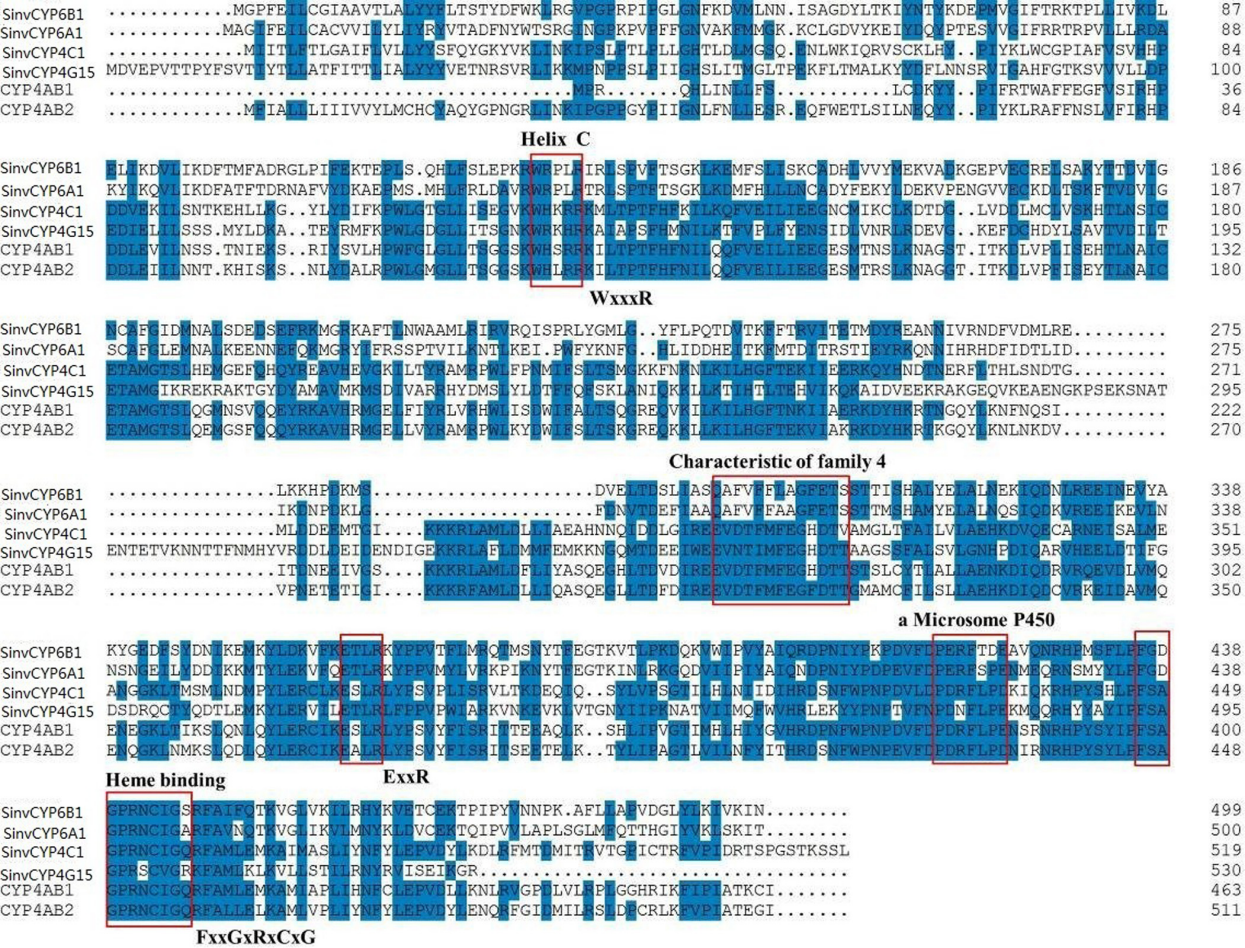
Alignment of the deduced amino acid sequences of SinvCYP6B1, SinvCYP6A1, SinvCYP4C1, and SinvCYP4G15 with CYP4AB1 and CYP4AB2 from the fire ant. Amino acid residues that were conserved among all four sequences and residues that were present in more than two P450 proteins are indicated by blue boxes. Invariant and highly conserved motifs in the P450 amino acid sequences are highlighted in red boxes.

The analysis of the putative amino acid sequences of SinvCYP6B1, SinvCYP6A1, SinvCYP4C1, and SinvCYP4G15 suggested that they had 5, 4, 8, and 6 transmembrane helices, respectively (http://www.ch.embnet.org/software/TMPRED-form.html). SinvCYP6A1, SinvCYP4C1, and SinvCYP4G15 were predicted to have no signal peptides on their N-termini (http://www.cbs.dtu.dk/services/SignalP/); however, SinvCYP6A1 was predicted to contain a single transmembrane domain from residues 2–24, SinvCYP4C1 was predicted to contain a single transmembrane domain from residues 2–21, and SinvCYP4G15 was predicted to contain two transmembrane domains from residues 10–32 and 45–64 at their N-termini (http://www.cbs.dtu.dk/services/TMHMM-2.0/). SinvCYP6B1 was predicted to have no signal peptide and no transmembrane domains at the N-terminus. The conserved C helix, the conserved I helix, the conserved K helix, the conserved sequence of a microsome P450 and the heme-binding domain are boxed in Fig 1.

### Phylogenetic relationships of *S*. *invicta* CYP6 and CYP4 proteins

**Fig 2:**
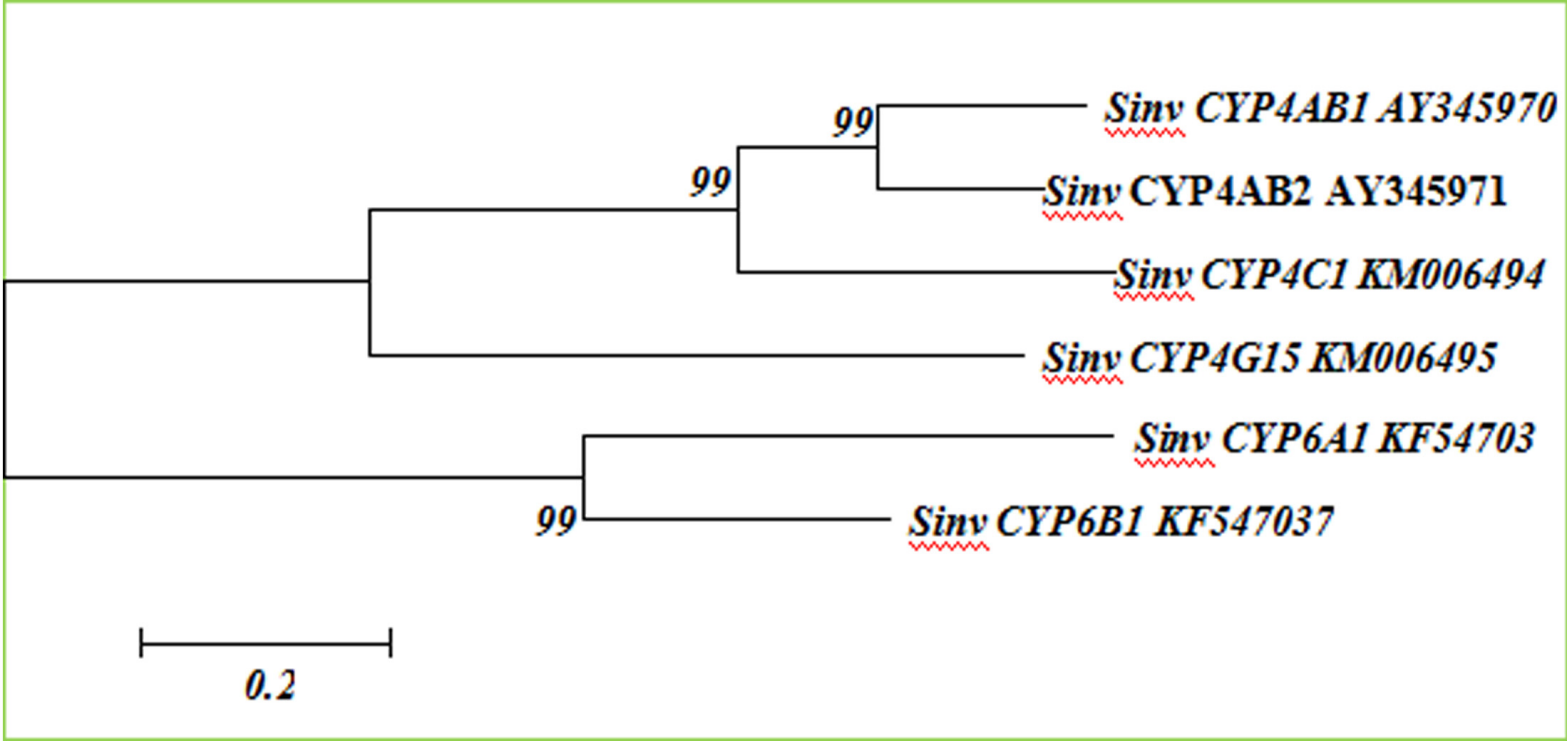
Phylogenetic relationships among the CYP4s and CYP6s from the fire ant. The CYPs are presented with their GenBank accession numbers. The un-rooted phylogenetic tree was constructed using the neighbor-joining method. Nodes indicate bootstrap values calculated with 1000 replicates.

In the phylogenetic tree, the complete amino acid sequence of SinvCYP4C1 exhibited high identity with the published sequences of the CYP4 families and shared 52.3% and 54.7% identity, respectively, with CYP4AB1 and CYP4AB2, which were grouped together. However, the SinvCYP4G15 sequence exhibited only a 31.2% identity with SinvCYP4C1. SinvCYP6B1 shared 51.1% identity with SinvCYP6A1, and they were grouped together (Fig 2).

We also investigated the evolutionary relationships of SinvCYP6B1 with the other insect CYP6 family sequences, SinvCYP4C1 and SinvCYP4G15 with the other insect CYP4 family sequences, and SinvCYP6B1 and SinvCYP6A1 with the other insect CYP6 family sequences (Fig A in S1 File). These sequences exhibited a relatively high identity with those of members of Hymenoptera, including *Apis mellifera*, *Bombus impatiens*, and *Nasonia vitripennis*; with members of Lepidoptera, including *Helicoverpa armigera* and *Helicoverpa zea*; and with members of Diptera, including *Musca domestica*, *Ceratitis capitata*, *Drosophila melanogaster*, and *Anopheles gambiae*. The sequences appeared to be more closely related to those in Hymenoptera and Lepidoptera than to those in the other insect orders.

### The induction of P450 gene expression in workers

**Fig 3:**
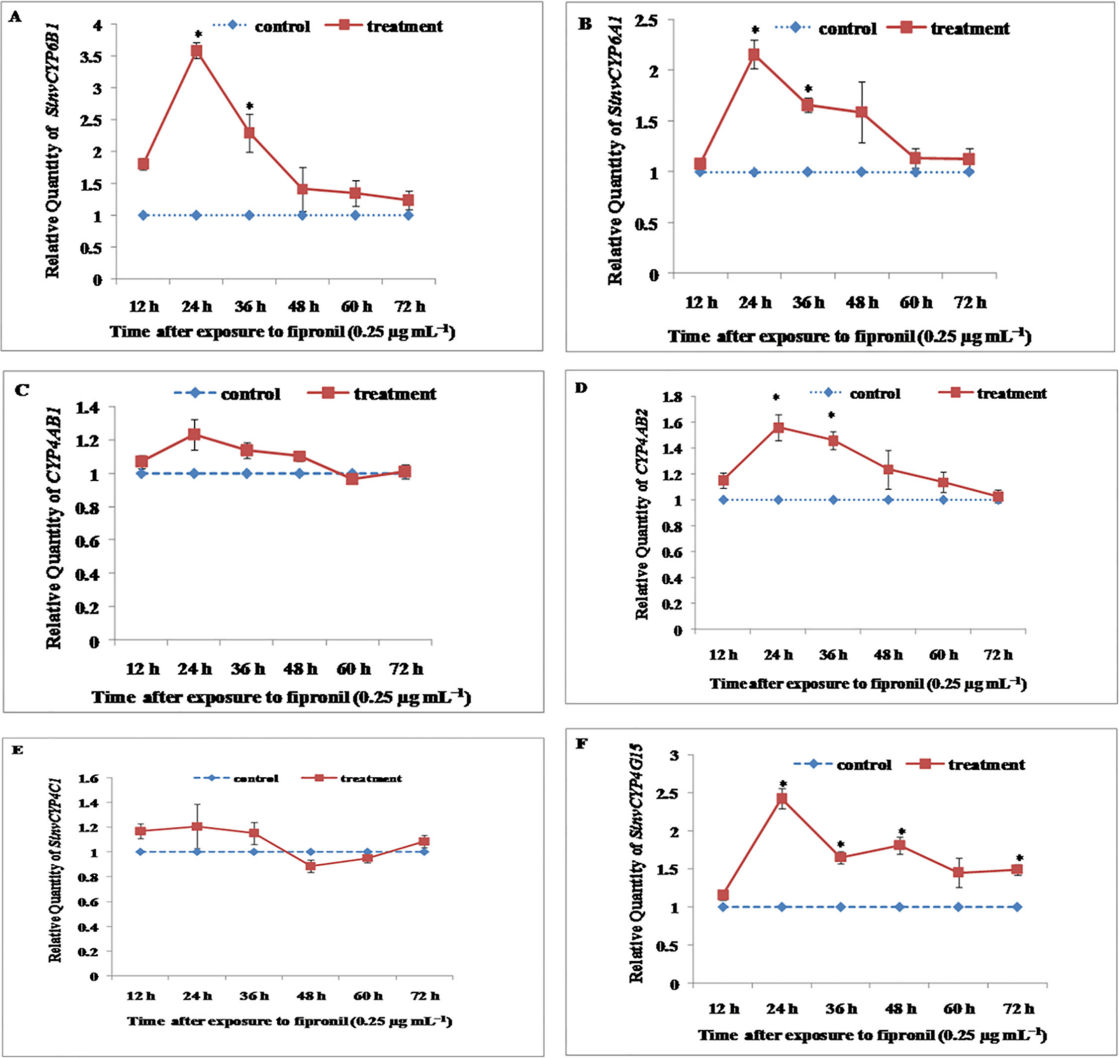
Relative expression levels of *SinvCYP6B1*, *SinvCYP6A1*, *SinvCYP4C1*, *SinvCYP4G15*, *CYP4AB1*, and *CYP4AB2* in workers of the fire ant following treatment with fipronil at the LC_50_ (0.25 μg mL^−1^). Relative expression levels of *SinvCYP6B1*, *SinvCYP6A1*, *SinvCYP4C1*, *SinvCYP4G15*, *CYP4AB1*, and *CYP4AB2* in workers following treatment with 0.25 μg mL^−1^ fipronil at 12, 24, 36, and 48 h (60 and 72 h) were determined by qRT-PCR. The experiments were repeated three times. The results are presented as the mean ± S.E. Significant differences within time points are indicated by *(P<0.05).

Compared with the controls, induction of four P450 genes, *SinvCYP6B1*, *SinvCYP6A1*, *CYP4AB2*, and *SinvCYP4G15*, in workers reached a maximum (3.6-, 2.2-, 1.6-, and 2.0-fold, respectively) at 24 h and then decreased 48 h after treatment with 0.25 μg mL^−1^ fipronil (Fig 3B, 3C, 3D, 3E and 3F). Nevertheless, no significant induction of *CYP4AB1* or *SinvCYP4C1* was identified in workers after treatment with fipronil at 0.25 μg mL^−1^ when compared with the controls (Fig 3C and 3E).

**Fig 4:**
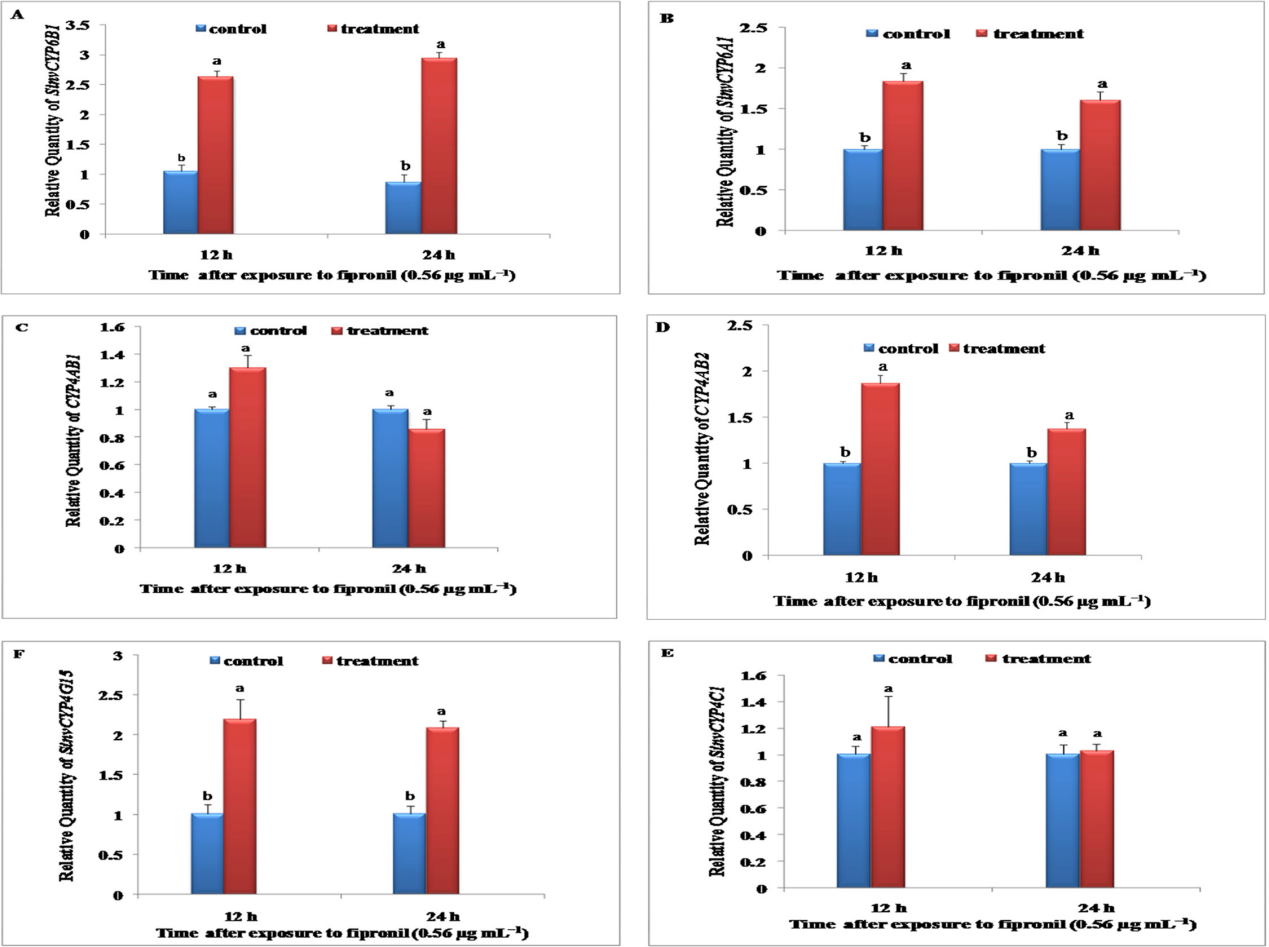
Relative expression levels of *SinvCYP6B1*, *SinvCYP6A1*, *SinvCYP4C1*, *SinvCYP4G15*, *CYP4AB1*, and *CYP4AB2* in workers of the fire ant following treatment with fipronil at the LC_90_ (0.56 μg mL^−1^). The relative expression levels of *SinvCYP6B1*, *SinvCYP6A1*, *SinvCYP4C1*, *SinvCYP4G15*, *CYP4AB1*, and *CYP4AB2* in workers following treatment with fipronil at 0.56 μg mL^−1^ at 12 and 24 h were determined by qRT-PCR. The experiments were repeated three times. The results are presented as the mean ± S.E. Significant differences within the same time point are indicated by different letters (P<0.05).

In the treatment with fipronil at 0.56 μg mL^−1^, the induction of three P450 genes, *SinvCYP6A1*, *CYP4AB2*, and *SinvCYP4G15*, in workers reached a maximum (1.83-, 1.6-, and 2.0-fold, respectively) at 12 h and then decreased after 24 h (Fig 4B, 4D and 4F) compared with the controls. The induction of *SinvCYP6B1* in workers reached a maximum (2.9-fold) at 24 h after treatment with fipronil at 0.56 μg mL^−1^ compared with the controls (Fig 4A), but there was no further significant induction caused by fipronil at 0.56 μg mL^−1^. Similarly, no significant induction in *CYP4AB1* or *SinvCYP4C1* was identified in workers after treatment with fipronil at 0.56 μg mL^−1^ compared with the controls (Fig 4C and 4E).

## Discussion

P450s have long been of particular interest because of their critical roles in the detoxification and/or activation of mutagens and xenobiotics, such as drugs, pesticides, plant toxins, and chemical carcinogens, and in the metabolism of endogenous compounds, such as hormones, fatty acids, and steroids [28–30]. In our current study, four novel P450 genes (*SinvCYP6B1*, *SinvCYP6A1*, *SinvCYP4C1*, and *SinvCYP4G15*) were isolated from workers of the fire ant. We also investigated the evolutionary relationships of *SinvCYP6B1* with the other insect *CYP6* family sequences, *SinvCYP4C1* and *SinvCYP4G15* with the other insect *CYP4* family sequences, and *SinvCYP6B1* and *SinvCYP6A1* with the other insect *CYP6* family sequences. These sequences exhibited relatively high identity with those of members of Hymenoptera, including *A*. *mellifera*, *Bombus impatiens*, and *Nasonia vitripennis*; with members of Lepidoptera, including *H*. *armigera* and *H*. *zea*; and with members of Diptera, including *M*. *domestica*, *Ceratitis capitata*, *D*. *melanogaster*, and *Anopheles gambiae*. The sequences appeared to be more closely related to those in Hymenoptera and Lepidoptera than to those in the other insect orders.

Our previous results revealed that the six P450 genes were overexpressed in workers, which was somewhat consistent with the overexpression of *CYP4AB1* and *CYP4AB2* mRNAs in workers compared with the expression in the other castes (e.g., queens) [31]. The gene expression patterns of P450 in different castes of the fire ant were related to the biological and physiological functions of P450. Moreover, the increase of O-demethylase activity in conjunction with the overexpression of the P450 genes in workers indicated that P450 in the fire ant might be involved in the detoxification of insecticides through constitutive overexpression [32]. This is largely consistent with the studies demonstrating that increased levels of P450 gene expression resulted in increased levels of total P450 [33,34]. This expression pattern suggests that the six P450 genes are potentially responsible for the detoxification of xenobiotic compounds, including insecticides or plant toxins, during feeding. In addition, further investigations with other eusocial insects (termites) suggest that P450 may also be important in the caste differentiation of social insects [35,36].

Tissue-specific expression of detoxification-related genes has been reported to potentially illustrate some of the functions of their biological and physiological roles [37]. As the insect midgut and fat body tissue are generally the primary organs of detoxification, they are the sites in which most of the insect P450s related to detoxification are expressed most highly [38]. To better understand the functions of the six P450 genes, the distribution of the six P450 genes in the tissues of workers was determined in our previous study. The results indicated that the high expression level of *SinvCYP6B1*, *SinvCYP6A1*, *SinvCYP4G15*, and *CYP4AB2* in the abdomens was observed, further indicating that these four P450 genes may be associated with detoxification of xenobiotic compounds (Fig B in S1 File). Furthermore, other tissues (e.g., the brain and nervous system) are important for the expression of the P450 genes and the development of insecticide resistance [39]. However, to accurately determine the functions of the six P450 genes in the detoxification of xenobiotic compounds, the tissue-specific expression levels of these genes after the workers exposure to insecticides should be further investigated.

The induction of the P450s by their substrates would reduce the effects of the substrates themselves by increasing their metabolism [40]. In our current study, the production of the *SinvCYP6B1*, *SinvCYP6A1*, *CYP4AB2*, and *SinvCYP4G15* transcripts was significantly induced by fipronil at the tested dosages and times. This finding indicates that multiple P450 genes may be involved in the detoxification of insecticides through the induction and constitutive overexpression of these detoxification genes. Similar results have been reported for *D*. *melanogaster* and *Culex quinquefasciatus* [34,41]. *CYP4G33* from *Chironomus tentans* can be induced by atrazine [42]. In addition, the production of the *SinvCYP4C1* and *CYP4AB2* transcripts was not significantly induced by fipronil at the tested dosages and times. This finding indicates that they may be not involved in the detoxification of insecticides through the induction of P450. *Diploptera punctata CYP4C7* was induced by ovarian hormone inducers [43].

Furthermore, in our previous study, exposure of workers to fipronil resulted in significant increases in the O-demethylase activity of P450, suggesting that in workers, P450 is a crucial detoxification enzyme involved in the metabolism of insecticides [32]. Huang et al. [44] reported that the specific activity of P450 in the larvae of *Chilo suppressalis* and *Sesamia inferens* decreased significantly after treatment with fipronil at a sublethal dose (LD_15_). Both the molecular and biochemical results indicate that *SinvCYP6B1* is strongly induced by exposure to fipronil, suggesting that *SinvCYP6B1* is a critical protein potentially associated with fipronil metabolism. Further studies should be performed to verify the function of *SinvCYP6B1* using RNAi to knock down the transcript level or to test the ability of *SinvCYP6B1* to metabolize fipronil in vitro.

Our study also found that P450 induction was dependent on dose and time. The low levels of induction at a high concentration (0.56 μg mL^−1^) might indicate a dysfunction of the induction system in insects exposed to high levels of poison. A lack of induction of P450 was also reported in *D*. *melanogaster* when the insects were challenged with insecticides at concentrations that exceeded the LC_99_ [45]. Thus, the highest level of induction occurred at a moderate concentration (LC_50_). The results for time dependence in workers after fipronil treatment are slightly different; after treatment with fipronil at 0.25 μg mL^−1^, the induction of most of the up-regulated P450 genes peaked at 24 h, whereas after treatment with fipronil at 0.56 μg mL^−1^, the induction of some of the up-regulated P450 genes peaked at 12 h. Our study indicates that the induction of the P450 genes leads to overexpression when workers are exposed to insecticides, resulting in an increase in the overall expression levels of multiple P450 genes in workers. In the development of insecticide resistance, the related P450 genes are expected to be expressed in the “first line of defense” tissues, such as the digestive system or the subcuticular fat body, which is the case for *CYP6A1* in *Drosophila* adults [46] and in house fly larvae [47]. However, further conclusions will require more extensive studies, such as the in vitro expression of a recombinant protein with subsequent examination of xenobiotic detoxification.

## Conclusion

This study provides evidence that *SinvCYP6B1*, *SinvCYP6A1*, *CYP4AB2*, and *SinvCYP4G15* are up-regulated in workers of the fire ant through constitutive overexpression and/or induction mechanisms of P450 genes, indicating their importance in metabolism of xenobiotics. Further studies are required to elucidate the function of these P450 genes in the fire ant. Our results are helpful for understanding the nature of P450s in this globally invasive pest.

## Supporting Information

S1 File **Fig A. Phylogenetic relationship of *S*. *invicta* (CYP6) with 15 CYP6s from other insects; *SinvCYP6B1* and *SinvCYP6A1* are boxed (A). Phylogenetic relationship of *S*. *invicta* (*SinvCYP4C1*) with 22 CYP4s from other insects; *SinvCYP4C1* is boxed (B). Phylogenetic relationship of *S*. *invicta* (*SinvCYP4G15*) with 22 CYP4s from other insects; *SinvCYP4G15* is boxed (C).** This un-rooted phylogenetic tree was constructed using the neighbor-joining method. Nodes indicate bootstrap values calculated with 1000 replicates. **S1 File. Fig B. Relative transcript levels of *SinvCYP6B1* and *SinvCYP4C1* in different tissues of workers as determined by qRT-PCR.** Each head, thorax, and abdomen sample contained material from 10 workers.
(ZIP)
