# Supplementary figures and images for: Cloning and Expression of Multiple Cytochrome P450 Genes: Induction by Fipronil in Workers of the Red Imported Fire Ant (Solenopsis invicta Buren)
Source: PLoS One. 2016 Mar 16;11(3):e0150915. doi: 10.1371/journal.pone.0150915 (PMC4794187; doi:10.1371/journal.pone.0150915)

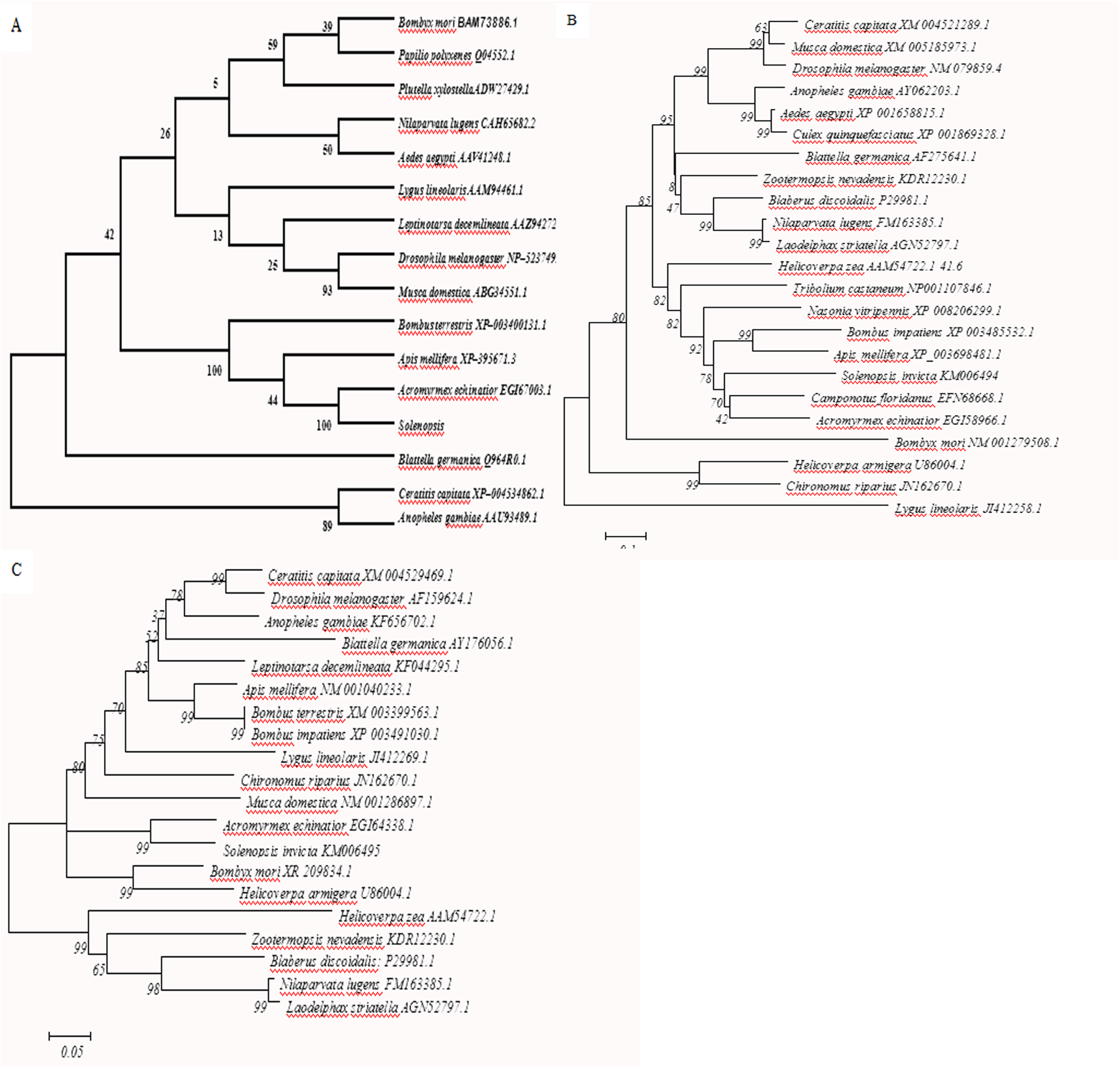

Supplement: S1 File — Fig A. Phylogenetic relationship of S. invicta (CYP6) with 15 CYP6s from other insects; SinvCYP6B1 and SinvCYP6A1 are boxed (A). Phylogenetic relationship of S. invicta (SinvCYP4C1) with 22 CYP4s from other insects; SinvCYP4C1 is boxed (B). Phylogenetic relationship of S. invicta (SinvCYP4G15) with 22 CYP4s from other insects; SinvCYP4G15 is boxed (C). This un-rooted phylogenetic tree was constructed using the neighbor-joining method. Nodes indicate bootstrap values calculated with 1000 replicates. S1 File. Fig B. Relative transcript levels of SinvCYP6B1 and SinvCYP4C1 in different tissues of workers as determined by qRT-PCR. Each head, thorax, and abdomen sample contained material from 10 workers. (ZIP) [file pone.0150915.s001.zip › Supporting information/Fig A.tif.tif]

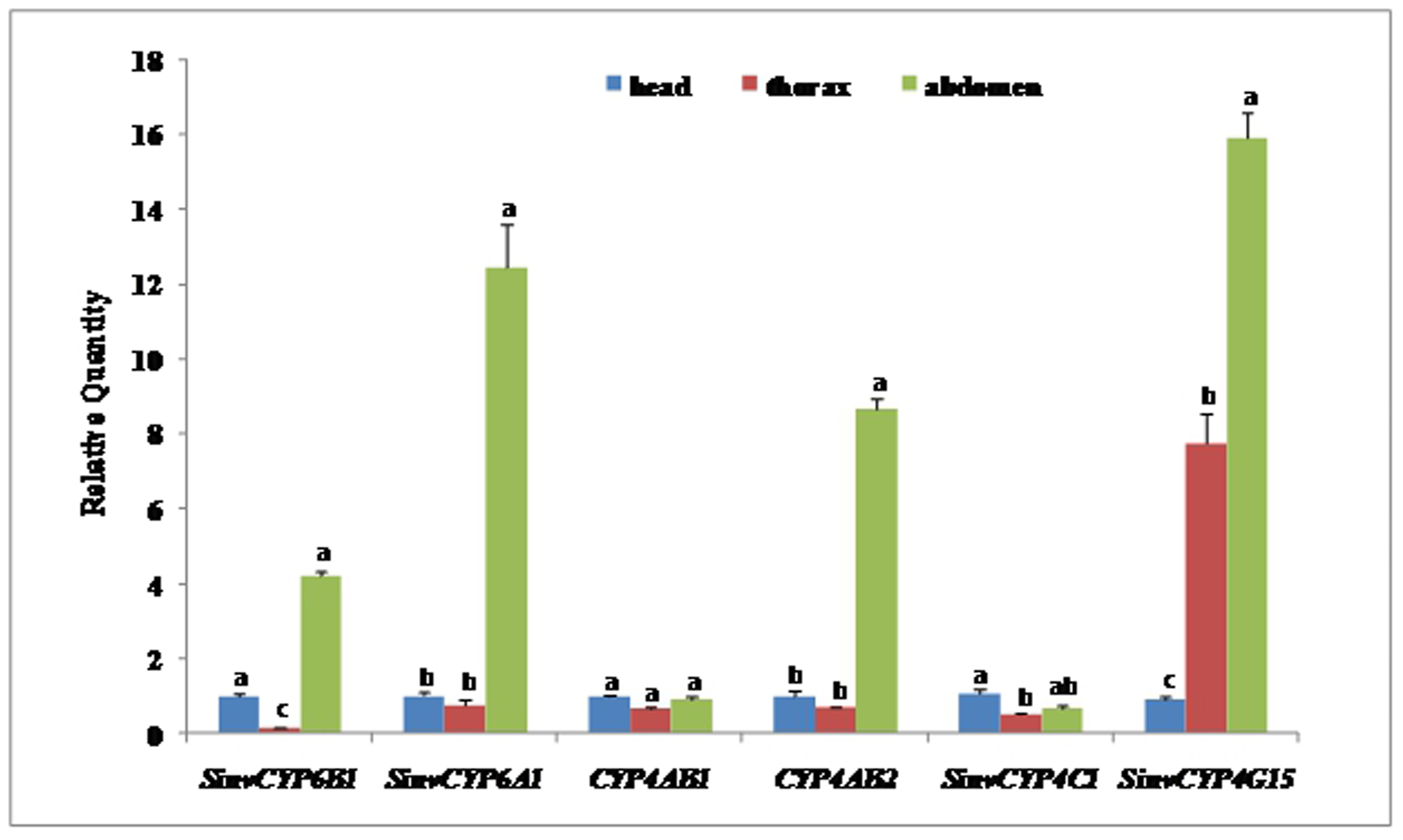

Supplement: S1 File — Fig A. Phylogenetic relationship of S. invicta (CYP6) with 15 CYP6s from other insects; SinvCYP6B1 and SinvCYP6A1 are boxed (A). Phylogenetic relationship of S. invicta (SinvCYP4C1) with 22 CYP4s from other insects; SinvCYP4C1 is boxed (B). Phylogenetic relationship of S. invicta (SinvCYP4G15) with 22 CYP4s from other insects; SinvCYP4G15 is boxed (C). This un-rooted phylogenetic tree was constructed using the neighbor-joining method. Nodes indicate bootstrap values calculated with 1000 replicates. S1 File. Fig B. Relative transcript levels of SinvCYP6B1 and SinvCYP4C1 in different tissues of workers as determined by qRT-PCR. Each head, thorax, and abdomen sample contained material from 10 workers. (ZIP) [file pone.0150915.s001.zip › Supporting information/Fig B.tif.tif]
